# Data on human neutrophil activation induced by pepducins with amino acid sequences derived from β2AR and CXCR4

**DOI:** 10.1016/j.dib.2016.05.065

**Published:** 2016-06-01

**Authors:** André Holdfeldt, Malene Winther, Michael Gabl, Claes Dahlgren, Huamei Forsman

**Affiliations:** Department of Rheumatology and Inflammation Research, University of Gothenburg, Gothenburg, Sweden

## Abstract

The data described here is related to the research article titled (Gabl et al., 2016) [1]. Pepducins with peptide sequence derived from one of the intracellular domains of a given G-protein coupled receptor (GPCR) can either activate or inhibit cell functions. Here we include data on human neutrophil function induced by pepducins derived from β2AR (ICL3-8) and CXCR4 (ATI-2341), respectively. ICL3-8 exerts neither direct activating effect on the NADPH-oxidase as measured by superoxide release nor inhibitory effect on FPR signaling. ATI-2341 dose-dependently triggers neutrophil activation and these cells were subsequently desensitized in their response to FPR2 specific agonists F2Pal_10_ and WKYMVM. Moreover, the ATI-2341 response is inhibited by PBP_10_ and the peptidomimetic Pam-(Lys-betaNSpe)6-NH2 (both are FPR2 specific inhibitors), but not to the FPR1 specific inhibitor cyclosporine H.

**Specification Table**

**Table:** 

Subject area	*Biology*
More specific subject area	*G-protein coupled receptor signaling*
Type of data	*Figures*
How was data acquired	*Isoluminol amplified chemiluminescence; luminometer*
Data format	*Processed*
Experimental factors	*Isolated human neutrophils were activated by pepducin or receptor specific agonists and the responses induced were determined*
Experimental features	*The effects of receptor specific inhibitors and desensitization profiles were determined*
Data source location	*Gothenburg, Sweden*
Data accessibility	*Data are within this article*

**Value of the data**

• Receptor specific neutrophil responses can be determined by the sensitive assay to measure superoxide production.
• The precise receptor involved can be identified by the desensitization profile and by the use of defined receptor specific antagonists.
• The data provide insights into the highly variable effects of pepducins which includes receptor hijacking.

## 1. Dat**a**

**Fig. 1:**
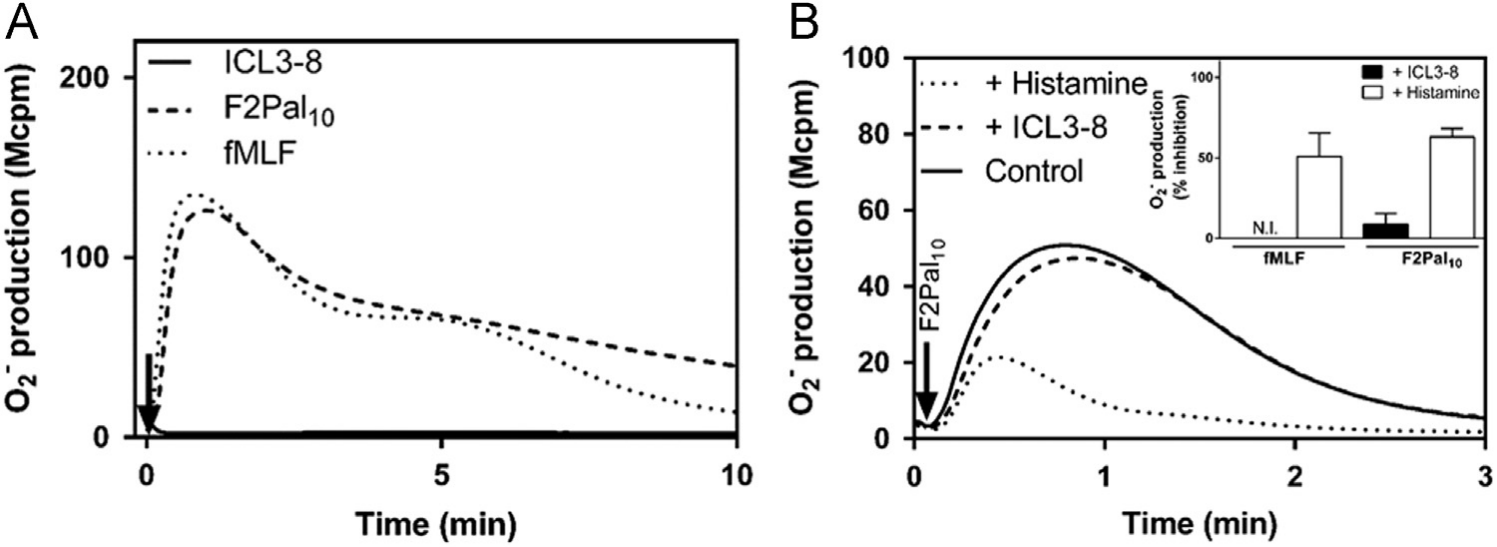
No direct activating or inhibitory effect on the FPR-mediated NADPH-oxidase response is induced by the Gs modulating pepducin ICL3-8. A) Human neutrophils (10^5^ cells) were pre-incubated with latrunculin A (25 ng/ml) at 37 °C for 5 min before the addition of the Gs modulating pepducin ICL3-8 (1 µM, solid line), the FPR1 specific agonist fMLF (100 nM, dotted line) and the FPR2 specific agonist F2Pal_10_ (1 µM, dashed line). Superoxide production was continuously measured by isoluminol-amplified chemiluminescence. The inset shows the response induced by the ICL3-8 pepducin with higher resolution. Representative curves out of at least five independent experiments using individual blood donors are shown. The arrows indicate the time points for addition of stimuli. B) Human neutrophils (10^5^ cells) were pre-treated with either ICL3-8 (1 µM) or histamine (10 µM) at 37 °C for 5 min before activation with F2Pal_10_ (1 µM; main figure and inset) or fMLF (100 nM; only inset). In the main figure, representative curves out of at least five independent experiments are shown. The arrow marks the time point for addition of F2Pal_10_. Inset: the inhibitory effect of ICL3-8 (black bars) and histamine (white bars) on FPR-mediated superoxide production (induced by the FPR1 agonist fMLF or the FPR2 agonist F2Pal_10_) expressed as percent inhibition compared to the activity induced in cells incubated without any inhibitor (mean±SD, *n*=4). N.I. equals no inhibition.

**Fig. 2:**
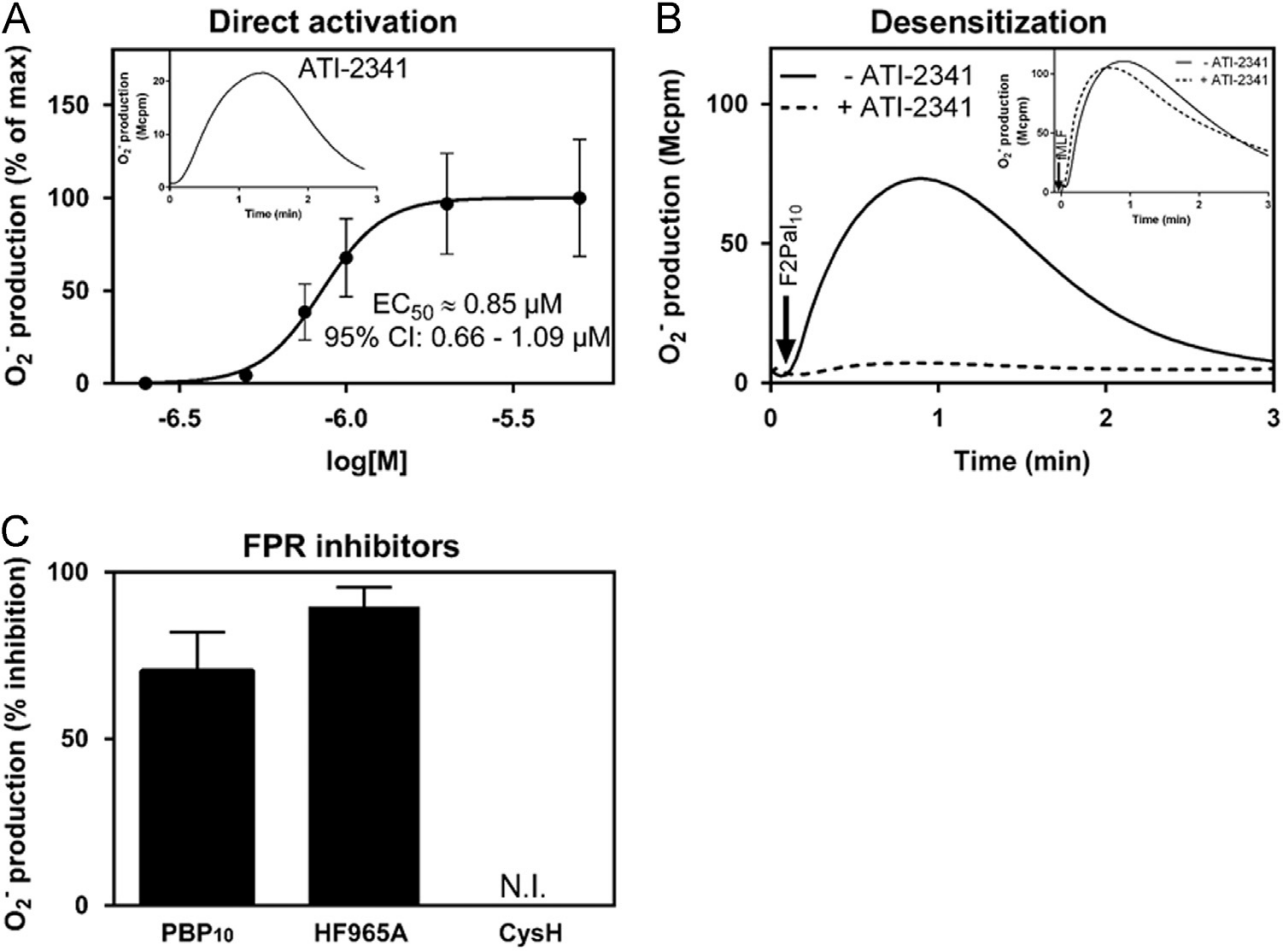
The CXCR4 pepducin ATI-2341 triggers a direct activation of the neutrophil NADPH-oxidase through FPR2. A) ATI-2341 dose-dependently triggers neutrophil superoxide production, as measured by isoluminol-amplified chemiluminescence. Data are presented as normalized peak response with the fitted curves. The EC_50_ value and the 95% confidence interval were calculated from 8 independent experiments. Inset: A representative ATI-2341 (1 µM) induced neutrophil response is shown. Abscissa, Time of study; Ordinate, Superoxide production (arbitrary units). B) Desensitized neutrophils incubated for 5 min at 37 °C without (solid lines) or with 1 µM ATI-2341 (dash lines) were activated with the FPR2 specific agonist F2Pal_10_ (1 µM; main figure) or the FPR1 specific agonist fMLF (100 nM, inset). Arrows indicate the time point for agonist addition. C) Neutrophils were pre-incubated for 5 min at 37 °C without any additive, with either of the two FPR2 specific inhibitors PBP10 (1 µM) or HF965A (1 µM), or with the FPR1 specific inhibitor cyclosporin H (CysH, 1 µM). The neutrophils were then activated with ATI-2341 (1 µM) and superoxide production was recorded by isoluminol-amplified chemiluminescence. The results are expressed as percent inhibition compared to the activity induced in cells incubated without any inhibitor (mean±SD, *n*=7). N.I. equals no inhibition.

Data describes human neutrophil activation, measured by isoluminol-enhanced chemiluminescence systems, with two pepducins derived from β2AR (ICL3-8) and CXCR4 (ATI-2341), respectively. Direct neutrophil activation by ICL3-8 and its modulatory effect on FPR signaling are shown (Fig. 1). In addition, data on dose-dependent neutrophil activation induced by ATI-2341 and the effects on this response of FPR specific agonists as well as antagonists are provided (Fig. 2).

## 2 Experimental design, material and methods

### 2.1 Neutrophil isolation

Human neutrophils were isolated from buffy coates as described [2], and diluted to 1×10^6^/ml in Krebs-Ringer phosphate buffer (KRG) containing glucose (10 mM), Mg^2+^(1.5 mM), and Ca^2+^(1 mM). The cells were kept on ice until use. Ethics approval was not needed since the buffy coats were provided anonymously and could not be traced back to a specific individual. This is in line with Swedish legislation section code 4§ 3p SFS 2003:460 (Lag om etikprövning av forskning som avser människor).

### 2.2 The NADPH-oxidase assay and receptor desensitization protocol

The release of superoxide anions from activated neutrophils was measured using isoluminol/HPR amplified chemiluminescence systems [3]. Vials with reaction mixtures of 900 µl containing isoluminol (20 µM), HRP (4U), neutrophils (1×10^5^/ml) with or without antagonist were incubated for 5 min at 37 °C. For receptor desensitization, cells were either activated or pre-incubated with receptor specific agonist for 5 min before stimulation. Stimuli (100 µl) were added and the light emission was recorded continuously.

### 2.3 GPCR pepducins and FPR specific ligands

The FPR2 pepducin F2Pal_10_ (Pal-KIHKKGMIKS, amino acid sequence from a part of third intracellular loop of FPR2), the β_2_AR pepducin ICL3-8 (Pal-LQKIDKSEGRFHV, amino acid sequence from a part of the third intracellular loop of β_2_AR) [4], and the CXCR4 pepducin ATI-2341 (Pal-MGYQKKLRSMTDKYRL, amino acid sequence from the first intracellular loop of CXCR4) [5] were from Caslo Laboratory (Lyngby, Denmark). All the other FPR1 and FPR2 specific ligands used have been described earlier [6].

## References

[1] Gabl M., Holdfeldt A., Winther M., Oprea T., Bylund J., Dahlgren C. (2016). A pepducin designed to modulate P2Y2R function interacts with FPR2 in human neutrophils and transfers ATP to an NADPH-oxidase-activating ligand through a receptor cross-talk mechanism. Biochim. Biophys. Acta.

[2] Boyum A., Lovhaug D., Tresland L., Nordlie E.M. (1991). Separation of leucocytes: improved cell purity by fine adjustments of gradient medium density and osmolality. Scand. J. Immunol..

[3] Bylund J., Bjornsdottir H., Sundqvist M., Karlsson A., Dahlgren C. (2014). Measurement of respiratory burst products, released or retained, during activation of professional phagocytes. Methods Mol. Biol..

[4] Carr R., Du Y., Quoyer J., Panettieri R.A., Janz J.M., Bouvier M. (2014). Development and characterization of pepducins as Gs-biased allosteric agonists. J. Biol. Chem..

[5] Quoyer J., Janz J.M., Luo J., Ren Y., Armando S., Lukashova V. (2013). Pepducin targeting the C-X-C chemokine receptor type 4 acts as a biased agonist favoring activation of the inhibitory G protein. Proc. Natl. Acad. Sci. USA.

[6] Dahlgren C., Gabl M., Holdfeldt A., Winther M., Forsman H. (2016). Basic characteristics of the neutrophil receptors that recognize formylated peptides, a danger-associated molecular pattern generated by bacteria and mitochondria. Biochem. Pharmacol..

